# 16 years follow-up evaluation of immediate vs delayed vs. combined hormonal therapy on fertility of patients with cryptorchidism: results of a longitudinal cohort study

**DOI:** 10.1186/s12958-022-00975-6

**Published:** 2022-07-14

**Authors:** Riccardo Bartoletti, Antonio Luigi Pastore, Filippo Menchini Fabris, Tommaso Di Vico, Riccardo Morganti, Andrea Mogorovich, Girolamo Morelli, Diego Peroni, Yazan Al Salhi, Alessandro Zucchi

**Affiliations:** 1 Department of Translational Research and New Technologies in Medicine and Surgery, University of Pisa, Pisa, Italy; 2 Urology Department, Sapienza University of Rome, ICOT Latina, Faculty of Pharmacy and Medicine, Corso della Repubblica 79, 04100 Latina, Italy; 3 Urology Unit, San Rossore Hospital, Pisa, Italy; 4 Department of Bio Statistics, Azienda Ospedaliera Universitaria Pisana, Pisa, Italy; 5 Urology Unit, Versilia Hospital, AO-Toscana Nord Ovest, Viareggio, Italy; 6 Department of Experimental and Clinical Medicine, University of Pisa, Pisa, Italy

**Keywords:** Cryptorchidism, Undescended testis, Hormonal therapy, Fertility, Gonadotropin

## Abstract

**Background:**

To investigate in a longitudinal cohort study, the best treatment to preserve fertility in cryptorchid subjects. Patients treated with immediate hormonal vs. delayed vs. combined (hormone plus surgery) therapy consecutively enrolled during the period 1987–1997, were evaluated.

**Methods:**

Two hundred fifty-five subjects were enrolled and 192 patients completed the follow-upt. One hundred fifty-six patients and 36 out 192 had monolateral and bilateral cryptorchidism, respectively. Twenty-nine out of 192 were previously treated by surgery alone (Group A), 93/192 by hormone therapy alone (Group B), 51/192 received sequential combined hormone therapy plus surgery (Group C) whilst 19/192 refused any type of treatment (Group D). The other 63 patients were considered lost to follow-up. All the patients underwent medical consultation, scrotal ultrasound scan, sperm analysis and Inhibin B, Follicular Stimulating Hormone (FSH) and Testosterone (T) serum level determination.

**Results:**

Testicular volume was found decreased in the Group D patients whilst hormone serum levels were comparable in all groups. Statistically significant differences for sperm characteristics were found in patients treated with hormonal therapy alone or combined with surgery (Groups B and C). These two groups reported better semen quality than patients who received surgery alone or no treatment. No differences were observed between monolateral and bilateral cryptorchidism patients.

**Conclusions:**

Early prolonged hormonal therapy is advisable in all patients with cryptorchidism independently from the surgical option of promoting testicular descent to the scrotum. Hormonal therapy provides in our study better chance to obtain adequate sperm quality in adult life.

## Introduction

Cryptorchidism represents one of the most common urogenital abnormalities in childhood and its incidence may be related to gestational age and newborn weight at birth, thus affecting 1.1–45% of preterm and/or < 2.5 kg neonates, and 1-4.6% of in term and/or > 2.5 kg infants [1]

Cryptorchidism is a consequence of abnormal testicular migration to the scrotum and the testis can be found in any place along its normal migration path [2]. It is bilateral in 30% of cases and should be considered in differential diagnosis with ectopy, retractile testis and anorchia [3, 4]. Approximately, 80% of all undescended testes are palpable along the course of the inguinal canal [5].

Undescended testes should be treated starting within the 18th month of life with the aim of avoiding risks of cosmetic, fertility or malignancy complications at long term follow up, though over the last 30 years the optimal timing for treatment has been a widely debated issue in the scientific world. Cryptorchidism treatment options include medical therapy and/or surgery [6]. Consensus recommendations for surgical management of cryptorchidism is defined by a recent literature review [7].

Cryptorchidism is described in one out of five azoospermic men * [8–10] with the hypothesis that reproductive function recovery could depend, rather than correct timing of treatment, on the involvement of one or both testes ° [11–13]. Undoubtedly bilateral cryptorchidism may be considered an augmented risk factor although some authors found that either follicle-stimulating hormone (FSH) and testicular volume or long-term sperm count were comparable among patients who had undergone mono or bilateral cryptorchidism, if treated in due time [14].

Hormonal treatment alone seems to be helpful for testicular descent but has a limited success rate (20–38%). Surgery, regardless of the timing in which it is performed, seems to be insufficient to determine a complete fertility recovery after treatment because it fails to address the underlying pathophysiological cause that consists in a defective mini puberty [15].

The objective of the present study is to evaluate the fertility of adult men who had previously undergone different treatment modalities for cryptorchidism during the period 1987–1997.

## Materials and methods

Two-hundred and fifty-five boys between 6 and 20 months of age (mean 13 ± 3.9) with mono or bilateral cryptorchidism who received endocrinological and urological counseling and/or treatment were consecutively enrolled during the period 1987–1997 in an observational longitudinal cohort study. IRB approval is not available because the study consists of a cohort of patients consecutively selected but not randomized in the respective treatment group due to their young age and followed up for over 16-years. Exclusion criteria were previous hormonal or surgical treatments, concomitant chronic diseases, and genetic disorders. Patients were originally selected after medical consultation and counseling concerning the different available treatment options and were consequently treated according to the patient family preference after they approved and signed an informed consent. The options presented were long lasting hormonal therapy, surgical orchidopexy, hormonal therapy followed by surgery when the first was ineffective in determining complete testicular descent into the scrotum. Surgical treatment was performed by the same surgeon during a two-day hospital stay whilst medical therapy was administered at home according to a predefined treatment schedule. For all patients treated with hormonal therapy, human Chorionic Gonadotropin (hCG) was injected at the dosage of 1000 Units for 6 weeks and a repeated cycle of therapy again after 1 month, varying the treatment schedule based on patient weight (twice per week for babies with a body weight < 12 Kg and three times per week for babies with a body weight > 12Kg). A small number of parents decided not to submit their child to any pharmacological or surgical treatment for the risk of developing related adverse events.

Thereafter, all patients enrolled were clinically followed with repeated periodical outpatient clinic visits every two months for the first two years and then once a year until complete development of the testis, at the age of 18. Syndromic cases, boys with mobile testes or local recurrence in subjects with previously already descended testes were excluded from the study.

Patients were divided into 4 different groups according to the type of treatment received: group A received surgery; group B received hormonal therapy; group C received a combined sequential hormonal and surgical treatment; group D decided not to receive any treatment.

Treatments were not randomized but were assigned on patient family preference as well as on the need to be switched to surgery if unresponsive to the hormonal treatment alone.

Patients assigned to group A chose to be immediately treated by surgery. The procedure was performed under general anaesthesia with an inguino-scrotal access to the inguinal canal, dissection of spermatic chord with lisis of cremasteric muscle and fixation of the testis at the scrotum wall. Complications were recorded according to the Clavien Dindo classification [16].

Patients assigned to group B received hormonal treatment, according to the previously described schedule. Adverse events and adverse reactions were registered and classified according to the North Bristol NHS trust classification and adequately treated if necessary [17].

Group C included patients who initially received hormonal therapy but did not have a definite testis allocation in the scrotum and had undergone further surgical treatment.

Group D included subjects who decided to avoid the risks related to any kind of treatment although properly informed of the potential consequences of this choice on the fertility of the patient and the increased risk of developing testicular cancer.

One hundred ninety-two out of 255 patients (76.4%) were available for further analyses concerning fertility. Testes location and size were recorded as well as the Follicular Stimulating Hormone (FSH), Testosterone (T) and Inhibin B circulating level. Patients were invited to collect semen for spermiogram testing at least 3 different times at a distance of at least one week between each test, recording as result the median value between the three samples taken.

Sperm analysis was conducted by two different biologists and information regarding sperm concentration, normal sperm cells number and sperm cell motility were collected according to the WHO 2010 international standards [18].

All data regarding the testis volume measurement were calculated by Prader’s orchidometer.

Patients included in the “no treatment” group received additional medical consultation and scrotal ultrasound scan in order to monitoring the risk of testicular cancer onset.

### Statistical analysis

Statistical analysis included the median values for the characteristics of patients included in different groups with different median follow-up periods.

Categorical data were described by absolute and relative frequency, continuous data by mean value and standard deviation. To compare quantitative factors (sperm concentration, progressive sperm motility, normal sperm morphology, inhibin B levels, FSH levels, testosterone levels and total testis volume) with different types of treatment (surgery, hormonal therapy, combined, no treatment).

ANOVA one-way was used followed by multiple comparisons with the Bonferroni method [19].

Significance was fixed at 0.05. All analyzes were carried out by SPSS v.26 technology.

#### Statement of ethics

IRB approval is not available because this study consists of an ***observational longitudinal cohort study*** of patients consecutively selected (1987–1997), but not randomized in the respective treatment group due to their young age with a follow-up of over 16 years.

## Results

One hundred and ninety-two patients out of 255 came to the monitoring visit. One hundred and fifty-six out of 192 patients originally had monolateral cryptorchidism and thirty-six bilateral cryptorchidism. Clinical evaluation and ultrasound, reported in the old medical records, showed the presence of undescended testis in the proximal third part of inguinal channel in 17% of patients, while 83% in the middle or proximal part of it.

Twenty-nine out of 192 patients were treated by surgery alone, 93 by hormone therapy, 51 received sequential combined hormone therapy plus surgery, and 19 refused any type of treatment. The other 63 patients were considered lost to follow up.

The median value of patients’ age at diagnosis was 1.16 ± 0.27 years whilst the median age at follow-up was 17 ± 1.7 years. Patients’ age at follow up was also analyzed by mean values and percentiles values distribution. Patients’ characteristics are described in Table 1.

**Table 1:** Characteristics of cryptorchid patients distributed in different groups according to the treatment received

Group	Pts.	Age at the time of first diagnosis and treatment median ± SD (years)	Monolateral N (%)	Bilateral N (%)	Age at follow up median ± SD (y)
Surgery	29	1,08 ± 0,24	25 (86)	4 (14)	16 ± 1,9
Hormonal	93	1,16 ± 0,27	75 (80)	18 (20)	17 ± 1,7
Combined	51	1,16 ± 0,28	41 (80)	10 (20)	16 ± 1,6
No treatment	19	1,00 ± 0,24	15 (78)	4 (22)	18 ± 1,4
Total	192	1,16 ± 0,27	156 (81)	36 (19)	17 ± 1,7

Results regarding sperm concentration, progressive sperm motility, normal sperm morphology, testicular volume as well as Inhibin B, Follicular Stimulating Hormon (FSH) and Testosterone (T) serum levels were collected and compared in the different groups by analysis of variance (Anova one way) and reported in Table 2.

**Table 2:** Analysis of Variance for serum hormone levels and sperm characteristics. Differences for sperm characteristics resulted statistically significant among patients treated with different treatment modalities as well as testicular volume variations whilst serum hormone levels remained comparable in all the groups

Analysis of variance (ANOVA one-way)					
Factor	Group	N	Mean	Standard deviation	*p*-value
Sperm concentration (mill/ml)	Surgery	29	19,72	14,15	< 0,0001
	Hormonal therapy	93	31,94	21,27	
	Combined	51	23,45	15,97	
	No treatment	19	7,53	3,85	
	Totale	192	25,42	19,34	
Progressive sperm motility (%)	Surgery	29	33,34	5,89	< 0,0001
	Hormonal therapy	93	50,28	13,07	
	Combined	51	38,92	5,83	
	No treatment	19	9,95	6,43	
	Totale	192	40,71	15,75	
Normal sperm morphology (%)	Surgery	29	3,83	1,73	< 0,0001
	Hormonal therapy	93	13,44	6,92	
	Combined	51	14,53	6,60	
	No treatment	19	1,74	1,45	
	Totale	192	11,12	7,59	
Inhibin B (pg/ml)	Surgery	29	189,45	70,02	0,063
	Hormonal therapy	93	211,76	46,02	
	Combined	51	211,55	67,40	
	No treatment	19	236,84	76,27	
	Totale	192	210,82	60,11	
FSH (IU/L)	Surgery	29	3,31	1,33	0,105
	Hormonal therapy	93	3,10	1,78	
	Combined	51	2,59	0,68	
	No treatment	19	3,26	1,45	
	Totale	192	3,01	1,48	
Testosterone (nmol/L)	Surgery	29	22,69	7,10	0,672
	Hormonal therapy	93	23,12	6,21	
	Combined	51	22,73	5,62	
	No treatment	19	21,21	5,15	
	Totale	192	22,76	6,08	
Total testis volume, US (ml)	Surgery	29	26,83	5,66	0,034
	Hormonal therapy	93	26,61	5,45	
	Combined	51	27,63	7,10	
	No treatment	19	22,95	4,75	
	Totale	192	26,55	6,00	

**Fig. 1:**
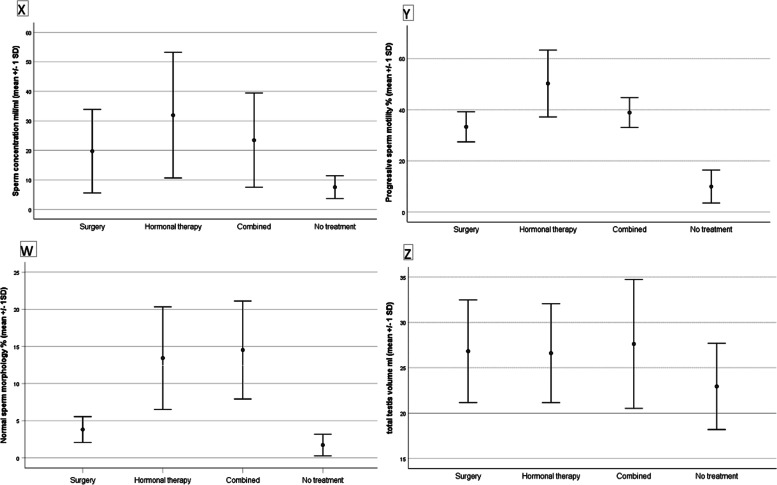
X) Sperm concentration Y) Progressive sperm motility W) normal sperm morphology and Z) testicular volume in the different groups of patients according to different treatment modalities. Statistically significant differences between hormonal treatment alone and combined hormonal therapy plus surgery vs. no treated patients were found for sperm concentration and normal sperm morphology. Conversely all the treatments adopted seemed to be efficient for progressive sperm motility maintenance. No significant differences for testicular volume were found between the groups

No significant differences were found among the groups for testicular volume, Inhibin B, FSH and T whilst differences in sperm characteristics were found to be significant. These differences were compared with Bonferroni methods (Fig. 1).

Patients who had previously been treated by hormonal treatment alone, reported the best results in terms of sperm concentration and progressive sperm motility. Furthermore, regarding normal sperm morphology and testicular volume, the study showed comparable results between hormonal treatment and combined treatment (hormone plus surgery) (Fig. 1). On the other hand, patients who had previously been treated by surgery alone showed regular testicular volume but worse sperm characteristics if compared to the groups of patients who previously received hormone therapy alone or combined sequential treatment. No significant differences were found between patients with monolateral and bilateral cryptorchidism probably due to the modest sample size.

Seminal assay was evaluated in all the patients according to the WHO reference values [18]. Seventeen out of 29 patients (57%) in group A showed normal sperm concentration and normal sperm motility but a lower rate (52%) of normal shaped spermatozoa. 91% of patients in group B had normal sperm concentration and motility and no patients with abnormal shaped spermatozoa. Intermediate results from the first two groups have been obtained in patients after sequential treatment with hormone therapy and surgery (group C). 71% of them had normal sperm concentration, 87% normal sperm motility and no patients had abnormal shaped spermatozoa. Only one patient of the “no treatment” group D had normal sperm concentration but deceptive results for sperm motility and normal shaped spermatozoa. Similarly, Inhibin B serum levels were found to be higher in the group of patients who refused any type of treatment in comparison to patients treated by hormonal therapy alone or in combination with surgery but comparable to the group of patients treated by surgery alone.

Sixteen out 19 patients who had been enrolled in the “no treatment” arm showed incomplete testes descent into the scrotum. The testes volume as well as the seminal parameters were substantially worse if compared to the other groups of patients in all 19 patients. However, hormonal levels were comparable and considered to be regular. No patients showed signs or suspicion of testicular malignancies at medical consultation or scrotal ultrasound scan.

Grade I-II adverse events of hormonal therapy, such as abnormal growth of the penis, skin erythema, penile erections, abnormal growth of pubic hair, augmented skin pigmentation, and mood alterations (mainly aggressiveness) were found in 67 out of 93 patients in group B (72%) and 40 out of 51 patients in group C (78.4%). The hormonal treatment was not suspended or interrupted in any patient. All data have been reported in Table 1. No adverse events were reported in group A.

## Discussion

Early treatment of cryptorchidism seems to be relevant in terms of fertility preservation, in maintaining a cosmetically satisfactory size of the testes and in preventing the risk of neoplastic transformation in adult age.

 European guidelines as well as Nordic countries guidelines recommend surgery within the first year of age or at the latest within the eighteen months of age [6, 20]. Schneuer et al. in a recent epidemiology report on 350,835 males who had previously undergone surgery for cryptorchidism within eighteen months of age, highlighted the need for assisted reproductive technologies during adulthood for all these patients. They reported that a 6-months delay for orchiopexy corresponds to a 5% increased risk of developing infertility and a 1% reduction in paternity [21].

The results obtained in the present series demonstrated that hormonal therapy associated to surgery seems to be able to determine comparative or improved results with respect to surgical treatment alone, although many patients reported low-mild adverse events. Hormonal therapy has been previously confirmed by other authors as mandatory in the treatment of incomplete testes descent because related to a systemic hormonal dysfunction [22].

The use of hormone therapy is still controversial in the current literature [3]. However, the association of hCG and intranasal LHRH seems to give the most consistent results in semen improvement especially in subjects with bilateral cryptorchidism. Side effects are more present during hCG therapy such as frequent erections, penile development, pain at the injection site and in the genital region. Cryptorchid testes are characterized by an abnormal testicular histology with a negative association between age and germ cell count except for patients who underwent surgery before 6 months of age who showed > 2.0 germ cells per tubulus (*p* < 0.0001) [22]. However, one out of three boys who early underwent surgery, presented an abnormal sperm count (< 40 × 10^6^) after 20 years and four of them had azoospermia [23]. Therefore, there is not a strong association between germ cell count and total sperm count thus demonstrating that a lack of adequate hormonal support or defective mini puberty in boys would confirm the theory that hormone therapy is necessary [15].

The results obtained in our longitudinal cohort study confirmed this theory. Only patients who received immediate hormonal therapy demonstrated statistically significant results in terms of sperm concentration, sperm motility and normal shaped sperm cells. Surgery remains the quicker solution to obtain cosmetic results and reduce the risk of further malignant testicular tissue transformation. Chandrasekharam et al. evaluated the effects of prepuberal hCG injections on the germ population and androgen production of adult rats and stated that the use of hCG in boys should have been critically re-evaluated [24]. The importance of an early hormonal treatment, independent of the effects on the testis descent, has been also highlighted by Hadziselimovic and Herzog showing improvement in seminal assay and confirmed by our results [25]. Patients who previously received hormonal treatments, obtained the best results in terms of fertility; those who had undergone prolonged and exclusive hormonal therapy demonstrated the best sperm quality in terms of both the number of spermatozoa per milliliter and the total motility at a high rate of morphologically normal cells. Combined hormonal therapy with surgery was also beneficial, although it includes a complementary surgical approach considered as more invasive for the patient. Group D patients or “no treatment” group presented slight sperm motility in terms of standard deviation probably due to the original position of the testes at early diagnosis of cryptorchidism. Data regarding the original position of the undescended testes are unavailable.

The time of exposure to hormones was related to the goal of obtaining a definite descent of the testes into the scrotum but not the preservation of function. The relationship between hormonal therapy and fertility preservation was not so clear at the beginning of the treatment. Surgery alone is clearly insufficient to determine a complete functional recovery although the timing for the procedure was adequate (12.8 ± 3.3 months). The time frame adopted for this retrospective analysis supported the theory that surgical procedure should have been performed within 24 months of life. All patients included in the present series were treated before the second year leading to an ethically correct treatment.

Hildorf et al. retrospectively evaluated testicular biopsies and hormone levels in patients who had undergone orchiopexy for cryptorchidism within the first year of life in the period between 2000 and 2019. They found that 25% of subjects with previous monolateral and 21% of bilateral cryptorchidism had a reduced number of germ cells, thus demonstrating that surgery is effective in obtaining a safe and immediate testicular descent but not a satisfactory fertility recovery [26]. Interestingly, no significant differences between patients with a mono or bilateral cryptorchidism were found; this is probably due to the limited number of subjects with bilateral cryptochidism although Barbotin et al. confirmed these data by analyzing a cohort of 223 with a history of cryptorchidism. They found no differences in follicle-stimulating hormone levels, testicular volumes, or sperm cells retrieval rates [14].

The main limitation of the study is represented by different number of subjects enrolled in each single group of treatment although at the time of patients’ enrollment each of the proposed treatment was considered as adequate to prevent further complications derived from cryptorchidism in the adult life.

 The “no treatment” arm should be considered as a limited option of treatment directly chosen by child’s parents after adequate medical counseling.

## Conclusions

In conclusion, undescended monolateral or bilateral testis may have a significant correlation to adult hormone levels and semen analysis. Our results partially confirm these data and highlight the need for early prolonged hormonal therapy in all patients with cryptorchidism aiming to increase the speed of testicular descent to the scrotum by surgery and consequently to maintain an adequate sperm quality in adult life.

Although semen quality showed a large variation among men with history of cryptorchidism, further research is needed to identify patients who are at largest risk of impaired semen quality when reaching adulthood.

## Data Availability

The datasets used and/or analysed during the current study available from the corresponding author on reasonable request.
